# Buckinghamshire Early Intervention Service’ (BEIS) Compliance With the National Institute for Health and Care Excellence (NICE) Antipsychotic Monitoring Guidelines: Practical Challenges, Including Those Posed by the Pandemic, in an Outpatient Setting

**DOI:** 10.1192/bjo.2022.495

**Published:** 2022-06-20

**Authors:** Shinn Tan, Harun Butt, Grace Pike, Busayo Williams, Alexandre Breton, Alaistair Reid

**Affiliations:** 1 Oxford Health, Oxford, United Kingdom; 2 Oxford Health, Aylesbury, United Kingdom

## Abstract

**Aims:**

Antipsychotic use is associated with cardiovascular and metabolic side-effects, which may contribute to increase mortality and morbidity in this patient group. This highlights the importance of physical health monitoring. We aimed to assess our compliance with the more stringent NICE guidelines, updated in September 2021.

**Methods:**

Half of BEIS team's caseload was audited (n = 67) during October 2021 for compliance with NICE's monitoring guidelines for patients initiated on antipsychotic medication. These included initial and, if indicated, repeat monitoring of body mass index (BMI), pulse, blood pressure (BP), blood results, electrocardiogram, and adverse effects. Patients who were not on antipsychotics were excluded. 61% of patients were initiated on antipsychotics as inpatients, and 39% were outpatients. These patients have been started on antipsychotics within the last three years. Data were collected via electronic record systems. 80% compliance was set as the standard, in line with National Clinical Audit of Psychosis standards.

**Results:**

In the first three months of antipsychotic initiation (61% as inpatients, 39% in the community) six out of nine parameters met standards (ranging from 2% to 100%), with BMI measurement (weekly), pulse and BP measurements and one month repeat haemoglobin A1C (HbA1c) failing. When only accounting for patients who were started on antipsychotics in outpatient settings (BEIS or crisis team), compliance was only met on two parameters.

Three months post initiation, when patients were mainly monitored in the community, only three of the nine parameters met compliance (lipids, HBA1c, and side-effects).

**Conclusion:**

Adherence to the NICE standards for physical health monitoring in the community poses significant challenges. Possible barriers include reduced patient contact during the pandemic, lack of awareness of monitoring requirements, poor documentation (particularly of declined screening) and a lack of time and resources. There is also a possibility of unnecessarily stringent and impractical guidelines which are difficult to achieve in outpatient settings – such as weekly BMI. We plan to implement interventions including providing a checklist for medical and nursing staff and encouraging patients to monitor their own blood pressure and weight at home. We will reaudit the same parameters in 6 months’ time.

